# Personality

**Published:** 1915-04

**Authors:** 


### The Department of Eugenics, Mental and Social Hygiene

CONDUCTED BY
DR. MALONE DUGGAN
813-814 Gibbs Building, San Antonio, Texas
All communications for this department should be sent to DR. DUGGAN

### Personality.

The interested visitor viewing the varied and magnificent buildings of Washington would be impressed with the air of distinctiveness and individuality expressed in the architecture. From the towering shaft of Washington's monument to the stately Capitol building there is seen and felt a definiteness of purpose in each and every structure. One is tall and majestic, commanding respect; another is massive and strong, inviting admiration; and another is artistic and magnificent, inspiring pleasure.

We ask why this difference, this distinctiveness of character? Let us analyze the reason. Each separate building was designed and built for a specific purpose, which is expressed in the fitness and adaptability of the materials used and in the form and strength of the structure. For example: The arched dome and stately halls of the Capitol give the impression of an atmosphere of learning and its suitableness as a temple of creative laws for a great nation. So, likewise, the magnificent and imperial reception rooms of the White House, with their imposing tapestries fittingly expresses the abode for the Chief Executive. Again, while each structure gives this impression of individuality and purpose, the observer instinctively regards each building as the integral part of a whole, but, at the same time, as being indispensable in its particular role. He recognizes the greater strength and beauty of one as compared with another, yet, he is not forgetful of the importance and usefulness of the lesser ones. In a similar manner the sky-scraper of our great cities pierces the clouds above the din of traffic and smiles complacently down on the smaller structures peering up below. Why so? It is because of the greater magnitude of the mortar, stone and steel which reared its walls above the rest. The sturdy oak in the fertile valley and the mammoth pine on the hillside bend their branches to the howling winds, and smile serenely at the admiration they command. Why so? It is because of the qualities of strength, fertility and endurance that makes their masterful position so sure. Why the emotions felt at viewing some great painting or sculpture? It is because of a creative genius that has stamped his being and thought on canvas and marble. Why the awe-inspiring pyramids that rear their dauntless heads above the tractless desert? It is because of the stupendous masonry of which they are composed that has stood unchallenged through the centuries. Why does one man achieve success over another; command respect and confidence; inspire courage and emulation beyond the possibility of his confrère? It is because of a psychic power peculiar to himself, inherent in him as a result of his personal development. It is this that we call personality; a distinctiveness of form, beauty and strength, a certain individuality that marks one thing from another, one person from another, and endows that thing or person with the recognition of respect, power or interest. Now, what is the explanation of this difference? Let us see. It is well in the province of scientific methods to base certain elementary phenomena on a hypothesis. For the sake of argument, then, let us assume (though the facts are regarded as already proven) that the self is composed of physical and mental activities. (We shall only deal in this discussion with the mental or psychic phenomena.)

Mental activities are composed of conscious and subconscious states. The organ of the body (the physical self) that is the basement of these activities, is the brain or the organ of the mind, and is fundamentally composed of universal ether. Matter we reduced to molecules, atoms, corpuscles and granules of ether. Spirit matter (product of mind) is an attenuated state of ordinary matter and is equally real with matter. It is represented as "material force," or the manifestation of the universal ether, and is further synthetised into energy and will. The universal ether is in a state of vibration and permeates all material realms and realities and embraces the sphere of the personal influence. Now, the self, through the mind, is capable of setting in vibration this ether that is within the body and sending it out beyond the limits of the body into the objective arena. In this way a certain region surrounding the individual is individualized and develops an atmosphere peculiar to that person.

It is thus seen that the brain is essentially a Marconi instrument capable of sending and receiving influences.

Ordinary communication between human minds obtains by means of the sense through material media; vibrations in matter induces the sensations of hearing, smelling, tasting, touching and seeing.

Mental energy is conveyed by means of invisible material media; it is transmitted when the medium is identical and transformed when it is different. The conscious and subconscious self alike can originate these ether vibrations.

The influences which are connected with your life are felt as ether vibrations. With some of these you are equally attuned, and when so attuned they appear prominent in the consciousness. With others you are only nearly attuned, and these can only be brought into consciousness by effort of will. It is this exercise of the will in bringing into consciousness, for judgment and action, the influences of daily experiences that gives growth and power to the personality.

But what is it that makes the difference between one personality and another? It is the difference in education of the self, and is determined by the quality of the environment and endowment of the individual. It is impossible for everyone to be equally strong in his personal influence. A man's capacity to be what he desires is limited by his heredity. His development within this limit depends upon his environments. By environment is meant his education and opportunity to make the best of this capacity. His success, therefore, is relative, and is not determined by any given standard, but by measuring up to his full capacity. Every man is equally successful, with any other man, who has fully developed to the limits of this endowment.

The force and strength of the personality so developed depends largely upon the force of will and the moral purpose which prompts the individual. No personality can be strong that is not grounded in moral principles and no influence can emanate from the individual until that value is first made a part of his own self. If he would desire to accomplish a certain purpose he must first make the object of his desire a part of himself. It will then become a material force capable of projecting itself to others. That is to say, he first assumes the object desired and by so doing the deeper self is instructed to form a corresponding habit. In time the object becomes a part of the inner self, and when so constantly asserted it finally becomes real. Thus it is that all the realities of life may be made to strengthen the personality. But this is not accomplished without effort and due regard for the mental processes involved. All habits and thoughts that are found injurious must be ignored and a constant atmosphere of honor, alertness and fidelity maintained. Invariable courtesy must always be observed and any unjust criticism avoided. The personal manner must be agreeable and friendly and a proper sense of reserve maintained.

The vibrations must be correspondingly strong, and a determined desire to win success consistent with the interest of others. The efforts must be persistent and there must be an unfailing harmony of motive and purpose. In the fullest realization of this power the person develops to a sense of oneness or harmony with the infinite.

So developed, the individual is, as it were, charged with dynamic force with the power of radiance, vibrating in the material media of universal ether, and influences the brain cells of all who came within his sphere of action.

Thus individualized, the man is master of himself, capable of accomplishing infinitely greater realities in his life and is a perfectly free being to do and act as he wills so long as such actions do not interfere with the rights of others.

